# Opportunities and Challenges for Improving the Productivity of Swamp Buffaloes in Southeastern Asia

**DOI:** 10.3389/fgene.2021.629861

**Published:** 2021-03-22

**Authors:** Paulene S. Pineda, Ester B. Flores, Jesus Rommel V. Herrera, Wai Yee Low

**Affiliations:** ^1^Philippine Carabao Center National Headquarters and Genepool, Science City of Muñoz, Philippines; ^2^Philippine Carabao Center at University of the Philippines – Los Baños, Laguna, Philippines; ^3^The Davies Research Centre, School of Animal and Veterinary Sciences, University of Adelaide, Adelaide, SA, Australia

**Keywords:** swamp buffalo, genomics, genetic improvement, genetic diversity, Southeast Asia agriculture

## Abstract

The swamp buffalo is a domesticated animal commonly found in Southeast Asia. It is a highly valued agricultural animal for smallholders, but the production of this species has unfortunately declined in recent decades due to rising farm mechanization. While swamp buffalo still plays a role in farmland cultivation, this species’ purposes has shifted from draft power to meat, milk, and hide production. The current status of swamp buffaloes in Southeast Asia is still understudied compared to its counterparts such as the riverine buffaloes and cattle. This review discusses the background of swamp buffalo, with an emphasis on recent work on this species in Southeast Asia, and associated genetics and genomics work such as cytogenetic studies, phylogeny, domestication and migration, genetic sequences and resources. Recent challenges to realize the potential of this species in the agriculture industry are also discussed. Limited genetic resource for swamp buffalo has called for more genomics work to be done on this species including decoding its genome. As the economy progresses and farm mechanization increases, research and development for swamp buffaloes are focused on enhancing its productivity through understanding the genetics of agriculturally important traits. The use of genomic markers is a powerful tool to efficiently utilize the potential of this animal for food security and animal conservation. Understanding its genetics and retaining and maximizing its adaptability to harsher environments are a strategic move for food security in poorer nations in Southeast Asia in the face of climate change.

## Introduction

The majority (~97%) of the 207 million buffalo population in the world is found in Asia, wherein about 20.51% are swamp buffaloes (FAOSTAT, 2018). There are two types of water buffaloes: swamp buffaloes and river buffaloes. Swamp buffaloes are mainly found in China and Southeast Asian countries. River buffaloes’ populations are larger than swamp buffaloes’ populations. They differ in chromosome number, phenotypic characteristics, and geographical locations, where they are usually found (Degrandi et al., 2014; Colli et al., 2018; Zhang et al., 2020).

Swamp buffaloes in Southeast Asia are raised by smallhold farmers because of their powerful draft capacity (OECD, 2017). This animal is utilized mostly for land cultivation; though it also provides milk, meat, hide, and horn, which are additional income sources to the farmers. However, due to increased farm mechanization, swamp buffalo have declined in value and its production has decreased by 4.92% for the last two decades (FAOSTAT, 2018). While swamp buffalo still holds a significant role in farmland cultivation, the purpose of this animal has shifted from draft power to meat and milk production.

One way to address the decline in production of swamp buffalo is to use genomic markers to selectively breed this animal for food security and conservation. Many countries in Southeast Asia have only started their breeding programs for swamp buffaloes in recent decades. Genetic improvement for buffalo in Thailand started in 1979 through their Department of Livestock Development. Genetic evaluation procedures, such as using estimated breeding values (EBVs), were conducted as part of their selection criteria for superior swamp buffaloes (Sanghuayphrai et al., 2013). Although genetic evaluation procedures are used in Thailand, breeding improvement and disease prevention are still lacking in some buffalo herds, leading to its low productivity, and hence highlight the need for upgraded buffalo management (Koobkaew et al., 2013; Sapapanan et al., 2013; Suphachavalit et al., 2013).

In the Philippines, a centralized research agency – Philippine Carabao Center (PCC) was established in 1992 to strengthen research and development on the Philippine carabaos. The PCC has several programs, such as the nationwide dispersal of semen for artificial insemination and bull loan programs, to upgrade buffaloes (Cruz, 2015). Cross breeding of the two types of water buffalo was carried out to improve the efficiency of the animal as their progeny showed increased body weight and milk production when compared to local swamp buffaloes. However, the crossbred progeny showed a decline in reproductivity, and hence backcrossing with a purebred swamp- or river-type was done to produce a ¾ Philippine swamp-type for draft power or ¾ river-type for dairy, respectively (Salas et al., 2000; Cruz, 2015). Genetic evaluation has also been done to select elite animals to improve milk traits in the Philippine dairy buffaloes (Herrera et al., 2018).

While there is no centralized agency exclusively for the development of water buffaloes in Malaysia, Indonesia, and Vietnam, regional efforts have been carried out to increase the performance of buffaloes in terms of reproductive performance, weight gain, and meat and milk production (Suryanto et al., 2002; Othman, 2014; Ariff et al., 2015). Buffalo management in Indonesia still follows the traditional approach leading to low productivity of the animal due to poor breeding plans, which has led to inbreeding within the population (Komariah et al., 2020). Despite breeding inefficiency, buffalo rearing by smallhold farmers is expected to contribute to the development of dairy industry in Indonesia. Vietnam produced and consumed more buffalo meat than beef; however, limited resources for research have stumped its intensified breeding program and buffalo development (Nguyen, 2000).

## Cytogenetics, Phylogeny, Domestication, and Migration

River and swamp buffaloes have 50 and 48 chromosomes, respectively. Although their chromosome numbers are dissimilar, these two sub-species can produce fertile offspring when crossed, which inherits 49 chromosomes due to the preserved characteristics of its chromosome arms (Degrandi et al., 2014). However, reproductivity is decreased in the hybrid progeny (Harisah et al., 1989; Borghese, 2011). This difference in chromosome number between the swamp and river buffalo is due to a tandem fusion translocation between river buffalo chromosomes 4 and 9 and swamp buffalo chromosome 1 (Di Berardino and Iannuzzi, 1981; Harisah et al., 1989), which was later confirmed when swamp buffalo genome assembly was made available (Luo et al., 2020). Studies on the karyotypes of swamp buffaloes that originated from the Philippines, Thailand, Malaysia, and Brazil showed conflicting results on the centromeres’ positions but they all agreed that the species has 48 chromosomes (Bondoc et al., 2002; Supanuam et al., 2012; Degrandi et al., 2014; Shaari et al., 2019). There are at least two possible reasons that account for differences in the centromeres’ positions: (1) different methods were used in the cytogenetic study (e.g., an addition of alcohol might have affected the arrangement of the chromosomes) and (2) subjective determination of each chromosomes’ centromere locations. Further investigation using a standardized method is needed to confirm the typical karyotype of swamp buffaloes.

**Figure 1:**
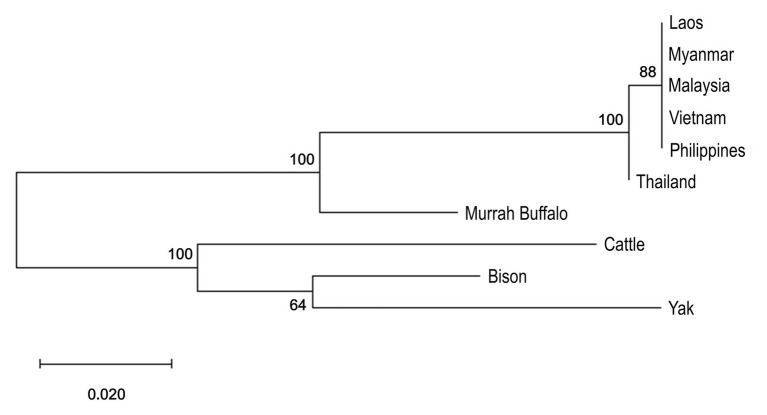
Phylogenetic analysis of mtDNA partial D-loop of swamp buffalo, Murrah buffalo, and three outgroup species was inferred by using a Maximum Likelihood method and a Tamura 3-parameter model in MEGA-X (Tamura, 1992; Kumar et al., 2018). Sequences were downloaded from the GenBank with the following accession numbers: Laos swamp buffalo (PopSet: 1174238592, KR008969-KR009068), Myanmar swamp buffalo (PopSet: 1174238592), Malaysia swamp buffalo (PopSet: 1605320276), Vietnam swamp buffalo (PopSet: 1174238592, 966874160), Philippines swamp buffalo (FJ873676-FJ873683), Thailand swamp buffalo (PopSet: 1174238592, KR008886-KR008939), Murrah buffalo – river-type buffalo (NC_049568), Cattle (NC_006853), American bison (NC_012346), and Yak (NC_006380). Initial trees were obtained by applying Neighbor-Join and BioNJ algorithms to a matrix of pairwise distances estimated using the Maximum Composite Likelihood (MCL) approach, and then selecting the topology with superior log likelihood value. One thousand bootstraps were done and their percentage values are displayed in the nodes.

Both river- and swamp-type have the same ancestral origin from wild Asiatic buffalo, *Bubalus arnee* (Cockrill, 1981). There is genetic separation for the two types of water buffaloes (Figure 1) and divergence between them is higher than the divergence observed between cattle subspecies (Yindee et al., 2010). Interestingly, comparison between river- and swamp-type buffaloes showed higher genetic variation within swamp populations despite the homogenous characteristics of their phenotypes and small number of breeds (Zhang et al., 2016; Paraguas et al., 2018; Sun et al., 2020b).

Divergence of the water buffalo to river- and swamp-type is estimated to have happened from 10 Kya to 1.7 Mya with the most probable period being from around 230 Kya or 900–860 Kya based on overlapping events such as geographical changes and concurrences from multiple studies (Tanaka et al., 1996; Wang et al., 2017; Sun et al., 2020a).

Swamp buffalo during post-domestication period followed two separate migration events from about 3,000 to 6,000 years ago in Asia (Wang et al., 2017). One was from Indochina border spreading around mainland China to the Philippines and the other was from mainland Southeast Asia and Southwest China border disseminating down to Indonesia (Zhang et al., 2016; Wang et al., 2017; Colli et al., 2018; Sun et al., 2020b). There is a genetically distinct population of swamp buffaloes in Southeast Asia that is thought to have arisen from the founder effect (Zhang et al., 2016; Colli et al., 2018; Sun et al., 2020b). A rare haplogroup was found in Thailand by Sun et al., 2020b using mtDNA D-loop sequences, which supported the hypothesis that Thai buffalo population may have come from an ancestral lineage (Colli et al., 2018). Considering that the wild Asiatic buffalo still exists in some parts of Thailand (Sarataphan et al., 2017), the ancestor of water buffalo may have also originated in mainland Southeast Asia (Lau et al., 1998).

## Genetic Sequence and Resource Availability

The whole genome sequence of a Mediterranean breed (UMD_CASPUR_WB_2.0) river buffalo was released in the NCBI in 2013 and published 4 years later (Williams et al., 2017; Table 1). A 90K SNP Buffalo Genotyping Array (Iamartino et al., 2013, 2017) has been available for use by researchers in the past few years; however, the SNP panel was created using a cattle reference genome (UMD3.1). The disadvantage of using the SNP panel for water buffalo is that it only represents 75% and 24.5% of the high quality, known polymorphic SNPs of river- and swamp-type buffaloes, respectively. The majority of the samples used in the SNP validation belonged to river buffalo, and hence a specific SNP panel for the swamp buffaloes is recommended since it is underrepresented in the 90K SNP Panel (Iamartino et al., 2013, 2017; Colli et al., 2018). Despite the limitation of missing some water buffalo specific SNPs, the genotyping array is still useful for genomic studies in river buffaloes but its usefulness remains limited in swamp buffalo (Herrera et al., 2016).

**Table 1:** Genome assemblies and resources available for water buffalo.

Assembly name	Genome size (Gb)	Contig N50 (kb)	Scaffold N50 (Mb)	Breed/origin	Type	Resources available for river and/or swamp buffaloes	References
UOA_WB_1	2.66	22441.5	117.2	Mediterranean	River	90K SNP Panel for buffaloes (Iamartino et al., 2013) – river and swamp buffalo Gene expression atlas (Young et al., 2019) – river buffalo Transcriptome resource (Williams et al., 2017) – river buffalo Intestinal microbial gene catalog (Zhang et al., 2017) – cattle and river buffalo Breeding programs Italy: Mediterranean breed – genetic improvement with genetic evaluation (http://www.anasb.it/) – river buffalo Brazil: Genetic improvement program (Bernardes, 2007) – river buffalo India: Genetic improvement and dispersal of semen from different breeds (Sahu et al., 2019) – river buffalo Pakistan: Genetic improvement of buffalo in Pakistan (GIBP; http://parc.gov.pk/index.php/en/faqy/131-narc/animal-sciences-institute/610-asi-breeding-genetics) – river buffalo Bangladesh: Buffalo Development Project (Hamid et al., 2017) – river buffalo	https://www.nature.com/articles/s41467-018-08260-0#citeas
Murrah_sire	2.62	9500.0	82.0	Murrah			River	https://www.biorxiv.org/content/10.1101/618785v2.full
Murrah_dam	2.62	5230.0	83.2	Murrah			River	https://www.biorxiv.org/content/10.1101/618785v2.full
GWHAAKA00000000	2.65	3100.0	116.1	Murrah			River	https://academic.oup.com/nsr/article/7/3/686/5737567
Bubbub1.0	2.77	25.0	7.0	Bangladesh			River	https://onlinelibrary.wiley.com/doi/full/10.1002/ece3.4965
UMD_CASPUR_WB_2.0	2.84	21.9	1.4	Mediteranean			River	https://academic.oup.com/gigascience/article/6/10/gix088/4101552
ASM299383v1	3.00	14.6	3.6	Egypt			River	Unpublished
Bubalus_bubalis_Jaffrabadi_v3.0	3.80	14.0	0.1	Jafarabadi			River	Unpublished
GWHAAJZ00000000	2.63	8800.0	117.3	Fuzhong	Swamp	Philippines: Genetic Improvement Program – upgrading and crossbreeding of river and swamp buffaloes (national dispersion of semen; https://www.pcc.gov.ph/genetic-improvement/) – river and swamp buffalo China: Upgrading and crossbreeding of river and swamp buffaloes (regional dispersion of semen; Yang et al., 2013) – river and swamp buffalo Thailand: BREEDPLAN program (analysis system developed in Australia; https://breedplan.une.edu.au/) – swamp buffalo	https://academic.oup.com/nsr/article/7/3/686/5737567

The scientific name for river buffalo is *Bubalus bubalis* and swamp buffalo is *Bubalus carabanesis*.

The river buffalo assembly based on the same animal used to create UMD_CASPUR_WB_2.0 was recently upgraded using long read sequencing for contig assembly and chromatin conformation capture technologies for scaffolding. The final assembly is called as UOA_WB_1 (Low et al., 2019) and is the best representative assembly of the river buffalo based on contiguity metric such as contig N50 (Table 1). The next assembly upgrade for the river buffalo will be a completely haplotype-resolved genome as demonstrated in cattle (Low et al., 2020). There are eight river buffalo assemblies but only one swamp genome assembly (Luo et al., 2020) in the literature and public databases. Besides genome assemblies and SNP panel, there are transcriptome resources that were used to create a large-scale gene expression atlas for the river buffalo and 3 million intestinal microbial gene catalogs from both buffalo and cattle (Williams et al., 2017; Zhang et al., 2017; Young et al., 2019).

## Comparisons Between River and Swamp Buffaloes

The latest river buffalo reference assembly (UOA_WB_1) is approximately 2.5 times more contiguous than the best swamp buffalo assembly (GWHAAJZ00000000) based on contig N50. Both of these assemblies benefited from long read PacBio sequencing to preserve assembly continuity and scaffolding with Hi-C reads has helped to produce chromosome-scale scaffolds. However, despite the availability of an impressive genome assembly, only about 0.76% of the submitted water buffalo nucleotide sequences were from swamp buffaloes in the GenBank as of January 2021. The river buffalo sequences represented the majority of water buffalo sequences in the public database. Additionally, there were only 17 genes for swamp-type, if one excluded the annotation from the recent swamp genome (Luo et al., 2020), which was a few magnitudes lower than the ~35,000 genes submitted for river-type buffaloes.^1^

Genomic regions that may be under selection have been analyzed in both swamp and river buffaloes. Interestingly, swamp buffaloes showed the signs of selection in docile behavior, muscle development, and fatigue resistance (Luo et al., 2020; Sun et al., 2020a). Among the genes under selection, *HDAC9* was found to be associated with muscle development in other species (Mei et al., 2019; Sun et al., 2020a). Luo et al. (2020) study on swamp buffalo genome also showed the expansion of *AMD1* gene that promotes muscle growth. This suggests the possibility of prospecting swamp buffaloes as a meat resource. Two critical starch digestion-enzyme genes, *AMY2B* and *SI*, were also identified that makes this species unique from other ruminants, which may suggest a new mechanism for adapting to rumen acidosis (Luo et al., 2020).

Signature of selection in river buffaloes showed over-representation in genes associated with immune-response, milk production, growth, and feed efficiency, which can be due to selection for milk production (Luo et al., 2020; Sun et al., 2020a). From the genes identified, *thyroglobulin* gene was associated with milk and meat quality traits, and was found to be a good candidate gene marker for meat marbling and milk fat percentage (Gan et al., 2008; Dubey et al., 2015).

Genetic variations in *DGAT1*, *MUC1*, *INSIG2*, and *GHR* in both river and swamp buffaloes were also associated with milk components, milk yield, and mastitis resistance, which are potential candidates for genetic selection (Deng et al., 2016; Li et al., 2018; da Rosa et al., 2020; El-Komy et al., 2020).

## Challenges and Opportunities

While Southeast Asian countries are experiencing improvements in agricultural productivity, it still remains relatively small (OECD, 2017). Considering the limited number of available genetic sequences and studies of swamp buffalo, it can be said that research funding allocation for this animal is low when compared to other bovine species. Countries from Southeast Asia should take a more progressive approach in studying the animal through genome science. Given the limited budget for research and development, this may be challenging as the costs for genomic research is high. Nevertheless, the trend of smaller farm sizes, increases in population and the effect of climate change, as well as agricultural innovations and developments, will likely push swamp buffalo farming toward intensified, profitable, and efficient farming (OECD, 2017).

Incorporation of genomic selection in genetic improvement programs has proven its success in dairy cattle and other livestock species, but which usually carried out in large-scale breeding programs and with intensive breeding selection (Sonstegard et al., 2001; Miller, 2010; Dekkers, 2012; Xu et al., 2020). On the contrary, local breeds are usually farmed in smaller population size and remain inferior in terms of productivity. Although the incorporation of genome science will maximize genetic gains of the animals, and hence an increase in productivity and income, the costs are relatively higher on a per animal basis (Iamartino et al., 2013; Biscarini et al., 2015). Despite the opportunities in breeding swamp buffaloes, economic constraints in smallhold farming remain a challenge for large scale and cost-effective genetic improvement programs (Biscarini et al., 2015; El Debaky et al., 2019). Nonetheless, the improvement of breeding stock through EBVs and proper management has shown significant increase in milk production in the Philippines, which demonstrated the value of systematic breeding programs for dairy buffalo (Flores et al., 2007). Rural farmers have seen buffalo rearing as a less risky source of income when compared to recurrent crop failures due to calamities such as typhoons and droughts (Escarcha et al., 2020). For example, through the support from government and organized groups, buffalo rearing holds the promise to enable sustainable living in smallhold farmers in the Philippines (Del Rosario and Vargas, 2013).

Genome editing (GE) technologies use zinc-finger nucleases, transcription activator-like effector nucleases and clustered regularly interspaced short palindromic repeats (CRISPR)/Cas9 to reproduce animals with economically important traits (Lee et al., 2020). It has been used in livestock species to produce polled (i.e., hornless) cattle (Young et al., 2020), mastitis resistant cows through insertion of *lysozyme* gene (Liu et al., 2014) and enhanced wool quality in goats and sheep by altering their *FGF5* gene (Hu et al., 2017; Li et al., 2017, 2019). The GE system has also been used to edit the swamp buffalo *GDF8* gene in cell line, which is a regulatory gene for myostatin that inhibits muscle development and differentiation (Su et al., 2018; Lee et al., 2020). Gene knockout of *GDF8* can increase the production of meat in cattle, goat, and sheep as double muscling was observed in experimental animals (Proudfoot et al., 2015; He et al., 2018; Wu et al., 2018; Ding et al., 2020). Examples of GE in water buffalo are limited but the opportunity to use this technology to enhance their economic traits remains to be explored. The applications of GE in livestock need to adhere to ethical standards and regulatory policies (McFarlane et al., 2019) that vary between countries. For example, the hornless cattle created using GE tools by the company Recombinetics was meant to proceed further in Brazil, but the plan was abandoned when unintended integration of plasmid was found in edited animals (Molteni, 2019; Norris et al., 2020). AquAdvantage salmon and GalSafe pigs are the only approved genetically modified animals for food specifically in United States and Canada (FDA, 2020).^2^ In Asia-Pacific region, it is unclear if livestock made using GE technologies will be acceptable in the near future (FAO, 2019).

Precision livestock farming (PLF) incorporates artificial intelligence technology to automatically monitor and manage animal production, predicts solutions for problems that may arise in the farm, and uses deep learning for genomic prediction (Banhazi et al., 2012; Pérez-Enciso and Zingaretti, 2019; Tullo et al., 2019). PLF assists large farms to be economically and environmentally sustainable; however, the cost of PLF still outweighs its efficiency for smallhold farmers (Hostiou et al., 2017; Carillo and Abeni, 2020). Genomic prediction using deep learning requires large datasets that are currently unavailable for the swamp buffalo. While PLF should be embraced in Southeast Asia, the limitation of high cost means its application to swamp buffalo farming remains infeasible in the near future.

Microbiome analysis for swamp buffaloes showed intrinsic difference to cattle microbiota that might explain buffalo’s efficiency in digesting fibers (Zhang et al., 2017; Iqbal et al., 2018). Rumen manipulation to reduce methane emission is also of interest in livestock management as it decreases the environmental impact of livestock production (Ungerfeld, 2018). In large-scale farmed populations, besides rumen related measurements, there are other low-cost proxies such as body weights and high-throughput milk mid-infrared that are also suitable to monitor methane emission (Negussie et al., 2017). Management and genetic improvement of swamp buffalo based on combination of these proxies may lead to production animals with less negative environmental footprint (Negussie et al., 2017; Ungerfeld, 2018).

With the increasing demand for food and mechanization in farming, swamp buffalo should be bred for meat and milk production through wide-scale or institutionalized development programs (Palacpac, 2010; Cruz, 2013). Buffaloes are well suited for tropical climate of Southeast Asia, and thus there is potential in upgrading local buffaloes to maximize milk production, which cannot be easily done with species maladapted to hotter and humid climates. Although swamp buffaloes are still susceptible to heat stress (Upadhyay et al., 2007; Rojas-Downing et al., 2017), their wallowing behavior and adaptability to warm conditions give them an advantage for hotter climate (Nardone et al., 2010).

## Conclusion

The potential of swamp buffaloes in food production is still untapped and genome research to increase its production is still limited. Understanding the capabilities of this species through a genomic approach can increase its productivity and benefit the farmers in the long run. The availability of high-quality swamp buffalo assembly is a leap forward in swamp buffalo genome science, because it opens up opportunities for technological advancement such as the creation of SNP panels specific to swamp buffalo for genetic improvement, diagnosis of diseases, and the study of genetic diversity. Although the cost of genomics is expensive and remains a challenge for developing countries in Southeast Asia, the opportunities to improve this animal for milk and meat production and animal conservation remain to be explored. With the rapid progress of technology and changing climates, rearing swamp buffaloes is a strategic option to increase smallhold farmers’ income. Breeding the animals through genomic selection is a good strategy to select meat and milk type swamp buffaloes while retaining its adaption to hotter, humid climates.

## Author Contributions

All authors contributed to the conception of the study, manuscript revision, read, and approved the submitted version. PP wrote the first draft of the manuscript.

### Conflict of Interest

The authors declare that the research was conducted in the absence of any commercial or financial relationships that could be construed as a potential conflict of interest.

## Footnotes

^1^https://www.ncbi.nlm.nih.gov

^2^https://aquabounty.com
